# Entropy, Assessed by Homeostatic Dysregulation on Electrocardiograms Predicts Fracture and Mortality

**DOI:** 10.1111/acel.70227

**Published:** 2025-09-10

**Authors:** Namki Hong, Sang Wouk Cho, Jungheui Kim, Hanjin Park, Dong‐Seon Kang, Seng Chan You, Hee Tae Yu, Kyoung Min Kim, Yumie Rhee, Alan A. Cohen, Steven R. Cummings

**Affiliations:** ^1^ Division of Endocrinology, Department of Internal Medicine, Severance Hospital Yonsei University College of Medicine Seoul South Korea; ^2^ San Francisco Coordinating Center California Pacific Medical Center Research Institute San Francisco California USA; ^3^ Department of Epidemiology and Biostatistics University of California San Francisco California USA; ^4^ Institute for Innovation in Digital Healthcare (IIDH) Yonsei University Health System Seoul Korea; ^5^ Department of Biomedical Systems Informatics Yonsei University College of Medicine Seoul South Korea; ^6^ Division of Cardiology, Department of Internal Medicine, Severance Hospital Yonsei University College of Medicine Seoul South Korea; ^7^ Division of Endocrinology, Department of Internal Medicine, Yongin Severance Hospital Yonsei University College of Medicine Yongin South Korea; ^8^ Department of Environmental Health Sciences, Butler Columbia Aging Center, Mailman School of Public Health Columbia University New York New York USA

**Keywords:** aging, electrocardiography, entropy, homeostasis, mortality, risk

## Abstract

Entropy, characterized by increased disorder throughout biological systems, can be quantified by homeostatic dysregulation (HD). One potential measure of HD is the dispersion of points from a normal value, approximated at the individual level by Mahalanobis distance (*D*_*M*_). We hypothesized that greater HD in electrocardiogram (ECG) would also reflect greater HD in the musculoskeletal system which, in turn, would be associated with age and manifest as an increased risk of fracture independently of age, bone mineral density (BMD), and history of fracture. We further hypothesized that greater ECG‐HD would be associated with increased risk of all‐cause mortality. A cohort of 7738 individuals aged 40 years or older who underwent a screening 12‐lead ECG between 2007 and 2018 was analyzed (mean age 63.5 years; 59.5% women; 5.5 years follow‐up). ECG‐HD was calculated as the natural log‐transformed *D*_*M*_ of five ECG measurements (ventricular rate, QRS duration, corrected QT interval, R axis, and T axis) referenced to young individuals (age 19–29). ECG‐HD increased with age (*r* = 0.28). Each standard deviation increment in ECG‐HD was associated with a 48% higher unadjusted fracture risk (HR 1.48, 95% CI 1.37–1.58) and remained significant after adjustment for clinical risk factors, ECG diagnoses, and femoral neck BMD (aHR 1.28, 95% CI 1.15–1.42). ECG‐HD was also associated with vertebral, nonvertebral, and hip fractures, and with mortality (aHR 1.44, 95% CI 1.18–1.74). ECG‐HD, a measurement of entropy in the cardiac system, was associated with fracture risk and mortality in adults, independent of clinical risk factors, BMD, and ECG diagnoses.

## Graphical abstract

Electrocardiogram‐based homeostatic dysregulation (ECG‐HD), a marker of cardiac conduction entropy, increases with age. Higher ECG‐HD is associated with a greater risk of aging outcomes including fractures and all‐cause mortality, independent of clinical risk factors, bone density, and ECG diagnoses.

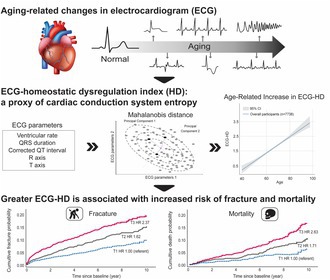

## 1 Introduction

Bone density declines and bone structure becomes weaker with aging (Steiger et al. 1992; Cawthon et al. 2009). These lead to an increased risk of fractures with aging (GBD 2019 Fracture Collaborators 2021; Ensrud 2013). However, studies have found that the association between age and risk of hip fracture, spine fracture, or major osteoporotic fractures remains significant after adjustments for bone density of the hip or spine (Ensrud et al. 2024; Langsetmo et al. 2023a, 2023b). This is also true for models that include measurements of bone structure by pQCT (Fink et al. 2018). This suggests that there are characteristics of bone that influence its strength besides geometry and density. Additionally, a history of fracture is a significant predictor of a future fracture at a different anatomical site after adjustment for bone density (Center et al. 2007).

A history of falls also predicts fracture independently of BMD (Harvey et al. 2018) and tests of physical and cognitive performance that decline with aging have also been associated with an increased risk of fracture (Bliuc et al. 2021; Cawthon et al. 2008). In concert, this constellation of findings suggests that there may be a property of bone strength and, perhaps, also of neuromuscular function, that worsens with age and contributes to an increased risk of fracture. As a property of more than one system, it may be a fundamental process of aging that occurs in many systems.

All matter becomes disordered with aging, a process known as entropy (Bortz 2nd 1986; Hershey and Lee III 1987). At a biochemical level, the entropy of biological tissues results from the gradual and random rearrangement or reactions of molecules (such as protein aggregation) that change the original structure of the matter (Pohl et al. 2012). Reactions that release energy may be essentially irreversible. Some of the molecular changes may also change the function of the matter if, for example, they impair the binding of substrate to an enzyme or change molecular configurations that contribute to essential structure (Gladyshev et al. 2021). The cumulative effect of molecular disorders is to change the functions and structure of cells and tissues with time, and higher‐order entropic processes such as incompletely healed wounds can also increase entropy at all biological scales (Gladyshev et al. 2021). Entropy is a familiar concept in physics and chemistry with established methods for its measurement (Clausius 1865; Boltzmann 2012). This fundamental and universal process, however, has not been well studied in research about tissues, such as the cardiac conducting system or bone, subjects of this investigation.

We postulated that the degree of entropy in one system would be associated with loss of function with aging in other systems. We used a measurement of multivariable statistical distance, Mahalanobis distance (*D*_*M*_) to test the specific hypothesis that the degree of variability or disorder in the cardiac conduction system, not solely attributable to a disease, would be associated with consequences of entropic disorder and dysfunction in bone and the neuromuscular system, with a consequent increased risk of fractures. More generally, we postulated that a greater degree of entropy in the cardiac conducting system would also be associated with similar changes in multiple systems, manifesting as an increased total mortality. Furthermore, we hypothesized that these associations, reflecting an underlying process of aging, would be attenuated by adjustments for age, but that because processes of aging such as entropy vary between individuals, the association would remain significantly associated with risks of fracture and mortality. We tested these hypotheses in a medical care cohort, VERTE‐X, that had routinely obtained electrocardiograms (ECGs) along with incident fractures and measurements of BMD.

## 2 Methods

### 2.1 Study Participants

**FIGURE 1:**
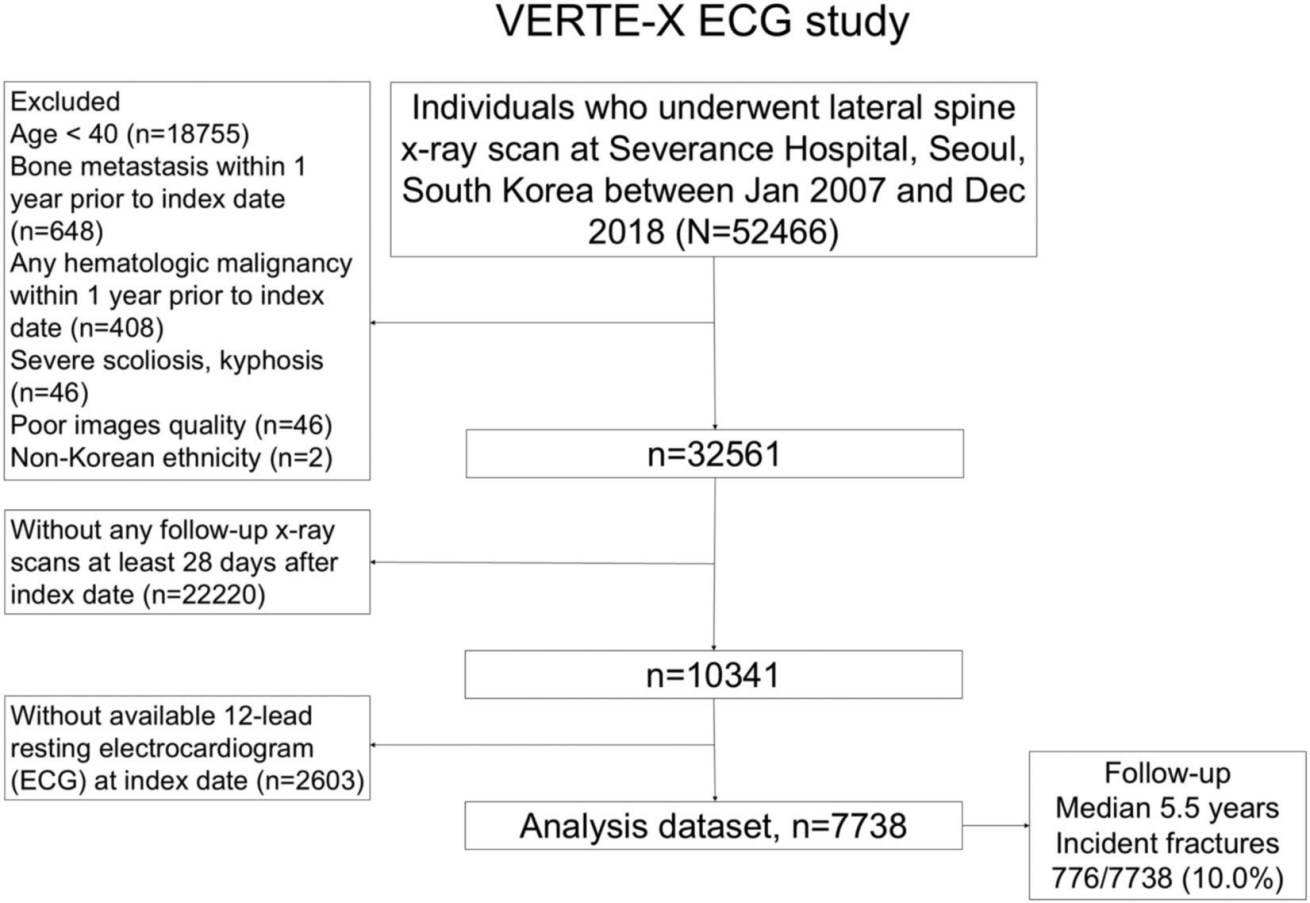
Study flow of the VERTE‐X ECG study.

VERTE‐X ECG is an ECG‐available subset of the VERTE‐X (VERTEbral fracture and osteoporosis detection in spine X‐ray) cohort (Figure 1). Details of the VERTE‐X cohort were published elsewhere (Hong et al. 2023). The purpose of the VERTE‐X study was to study the association of predictors including prevalent vertebral fracture with incident fracture and mortality. Demographic, clinical, and radiographic data of individuals aged 40 years or older who underwent spine radiograph at Severance Hospital, Seoul, South Korea between January 2007 and December 2018 were collected as baseline, then the occurrence of outcomes was followed up until the last observation date (December 31, 2023; Figure 1). Among a total of 10,341 participants in the VERTE‐X cohort, individuals with available 12‐lead resting ECG data taken on the cohort entry date were included in the VERTE‐X ECG study (*n* = 7738). Requirement of written informed consent was waived by the Institutional Review Board of the Severance Hospital (IRB no. 4‐2021‐0937).

### 2.2 ECG‐Based Homeostatic Dysregulation Index

**FIGURE 2:**
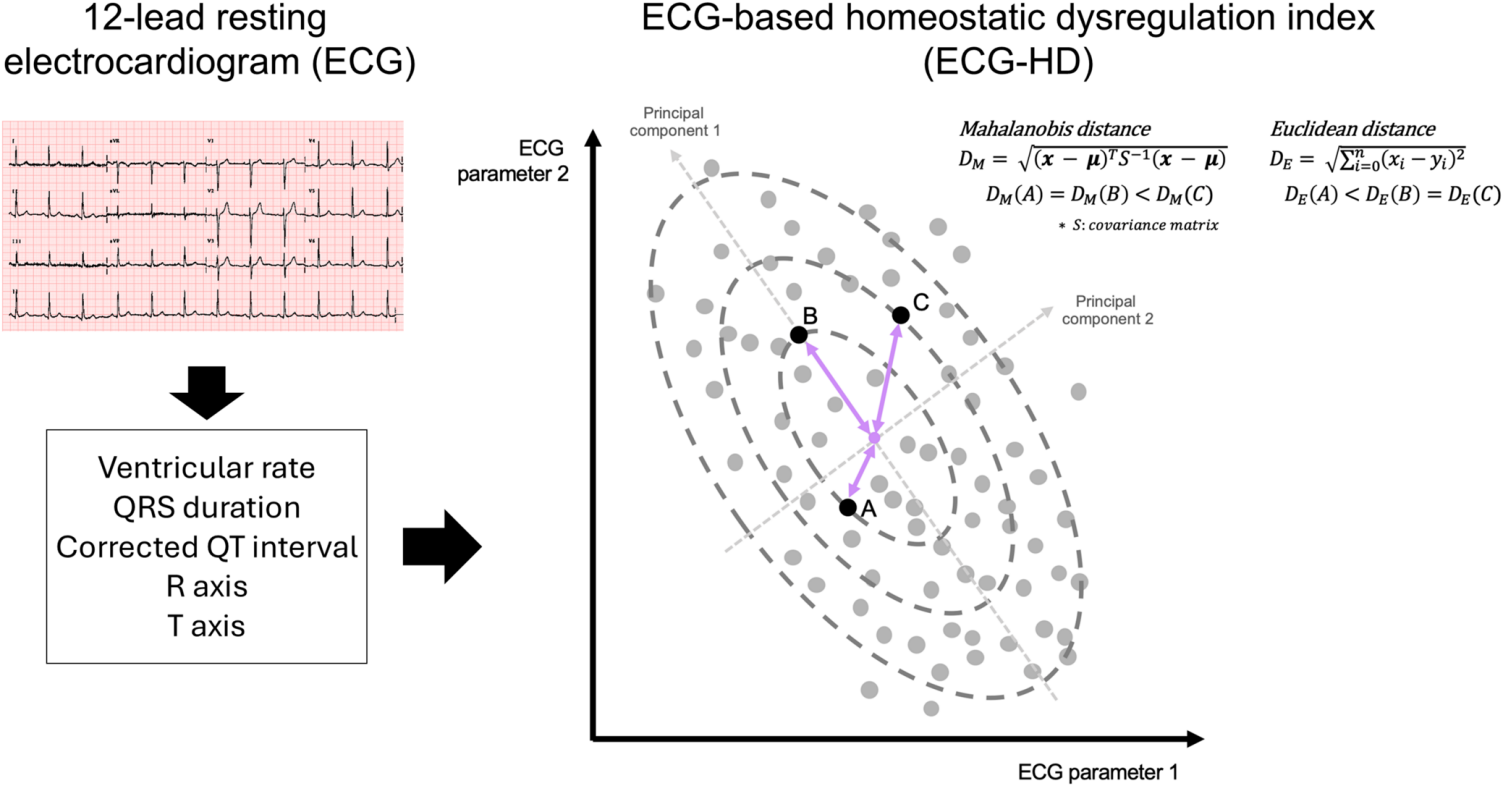
Conceptual diagram of ECG‐based homeostatic dysregulation index.

The standard 12‐lead ECG data was extracted from GE Healthcare's MUSE cardiology Information system (Figure 2). The ECG database included raw waveforms, measurement data, and automated ECG diagnosis statements generated by the built‐in software. A total of five ECG measurements (ventricular rate, QRS duration, corrected QT interval, R axis, and T axis) were used to calculate *D*_*M*_ from the centroid (normal state or low entropy state) for all participants, indicating how far each individual's ECG profile deviated from the baseline healthy population mean (Figure 2) (Mahalanobis 2018). PR interval and P axis were not included in the analyses due to potential missing values. Natural log‐transformed ECG‐derived *D*_*M*_ was defined as ECG homeostatic dysregulation index (ECG‐HD). The method of *D*_*M*_ as a homeostatic dysregulation index has been established in blood biomarkers to prove the association of dysregulation in multiple systems with various aging outcomes (Cohen et al. 2013; Li et al. 2015). To define the centroid data point to calculate ECG *D*_*M*_, we used an independent ECG dataset obtained from 37,848 individuals aged 19–92 who underwent health examination at Severance health checkup center between October 2014 and May 2021 (checkup ECG study, Figure S1). Mean value of ECG measurements in young individuals aged 19–29 were set as centroid (Table S1), while the mean of the overall population was set as an additional centroid for sensitivity analysis.

### 2.3 ECG Diagnoses

All automated ECG diagnoses generated by the GE built‐in software were manually reviewed and verified by two expert cardiologists (H.J. Park and D.‐S. Kang). ECG diagnoses were then annotated into diagnostic, rhythm, and form statements in accordance with the SCP‐ECG (CEN/ENC 1064 Standard Communication Protocol for computer assisted Electrocardiography) and PTB‐XL classification (Rubel et al. 2021; Wagner et al. 2020). To create mutually exclusive categories within diagnostic, rhythm, and form statements, we refined the classification as follows: diagnostics (6 categories; normal ECG [referent], conduction disturbance, hypertrophy, myocardial infarction, ST/T change, and two or more abnormalities); rhythm (5 categories; normal sinus rhythm [referent], sinus bradycardia, sinus tachycardia, atrial fibrillation, and other); and form (4 categories; normal form [referent], non‐specific ST changes, ventricular premature complex, and other; Table S2). For rhythm statements, 26 individuals (0.3%) had overlapping conditions (sinus bradycardia and other) and they were classified as “other” For form statements, 31 individuals (0.2%) had simultaneous ventricular premature complex and non‐specific ST changes. They were adjudicated to the ventricular premature complex category.

### 2.4 Covariates

To define covariates, a look‐back period was applied from January 1, 2004 (the start of available EHR data in the institution) up to each individual's cohort entry date between January 2007 and December 2018. For each individual, the look‐back window began from the date of the first available medical record within the period (median look‐back period 53 months [interquartile range 5–113 months]). Previous history of fracture was defined as the presence of either any morphologic vertebral fracture annotated by the algorithm‐based qualitative (ABQ) method in the baseline spine radiograph or clinical history of fracture at the spine, hip, wrist, upper arm, pelvis, and lower leg (Jiang et al. 2004). Chronic glucocorticoid use was defined as consecutive use of oral glucocorticoids (prednisolone‐equivalent dose ≥ 5 mg per day) longer than 3 months in the past 6 months from the index date. DXA areal bone mineral density (BMD) measurement (Discovery W and A, Hologic, USA) was collected as the measurement closest to the index date within a 6‐month observation window before or after the cohort entry date. DXA BMD was available in 76% (5859/7738) of the VERTE‐X ECG cohort. Femoral neck BMD T‐score was calculated using the NHANES III young White female reference (Looker et al. 1997). Comorbidity was assessed using the Charlson comorbidity index at the time of the index date, based on the ICD‐10 coding algorithm by Quan et al. (2005), mapped to KCD codes used in Korea. The Charlson comorbidity index was dichotomized at ≥ 2 to define individuals with multiple comorbidities (Walter et al. 2001).

### 2.5 Outcome Definitions

The primary outcome of the study was the occurrence of any incident fracture at the spine (morphologic or clinical), hip, distal forearm, upper arm, pelvis, or lower leg during the follow‐up period. The occurrence of the event was identified and ascertained by diagnostic codes, procedure codes, and medical records in the electronic health system of the institution. The ABQ method was used to define the new‐onset morphologic vertebral fracture in follow‐up radiographs compared to baseline (Jiang et al. 2004). Secondary outcomes included incident vertebral fracture (morphologic or clinical), nonvertebral fracture, major osteoporotic fracture (vertebral, hip, distal forearm, or upper arm), hip fracture, and death. The date of mortality was ascertained by the review of issued death certificates. Outcome data were ascertained through hospital records up to December 31, 2023. For fracture outcomes, follow‐up time was defined as the duration from cohort entry to the occurrence of a fracture or censoring at the date of last known contact (i.e., outpatient visit, hospital discharge, or death), whichever occurred first. For death as the outcome, follow‐up time was defined as the duration from cohort entry to the occurrence of death or censoring at the date of last known contact (i.e., outpatient visit or hospital discharge).

### 2.6 Statistical Analysis

Clinical characteristics of study participants were presented with the comparison between groups with or without incident fracture using two‐sample independent *t*‐test and chi‐squared test for continuous and categorical variables, respectively. All ECG measurements were normalized as stratified by sex using the mean and standard deviation of the reference population prior to the calculation of *D*_*M*_. *D*_*M*_ was calculated using the “mahascore” command of Stata. The association between ECG‐HD and age was plotted using fractional polynomial regression curve (Royston and Altman 1994). One‐way analysis of variance (ANOVA) with Bonferroni multiple testing correction was used to analyze the difference of ECG‐HD values by categories of ECG diagnoses. Kaplan–Meier failure curves for incident fracture and mortality were plotted by tertile groups of ECG‐HD. Cox proportional hazard models were built to assess the association between ECG‐HD and outcomes with adjustment for clinical risk factors (age, sex, body mass index, previous history of fracture, and chronic glucocorticoid use), ECG diagnoses, and femoral neck BMD. In multivariable model 4, which included femoral neck BMD as an adjustment variable, individuals with missing BMD values were excluded from the analysis. Competing risk between overall fracture and mortality was investigated by the Fine and Gray proportional subdistribution hazard model (Fine and Gray 1999). No violation of the proportional hazard assumption was observed. The statistical significance threshold was set at two‐sided *α* = 0.05. All statistical analyses were performed using Stata 18.0 (Statacorp, Tx, USA).

## 3 Results

### 3.1 Characteristics of Study Participants

Data of 7738 participants (mean age 63.5 years; 59.5% women) in the VERTE‐X ECG study were analyzed (Table 1). Median follow‐up duration was 5.5 years with interquartile range 3.1–7.7 years, with the occurrence of incident fracture in 776 participants (10.0%) during follow‐up (Figure 1). Individuals with incident fracture had a higher prevalence of older age, women, previous fracture history, chronic glucocorticoid use, and a high CCI score. Compared to individuals without any incident fracture, those with incident fractures had a higher ventricular rate, shorter QRS duration, longer corrected QT interval, and more left deviation of the R axis (Table 1). For ECG diagnoses, 68% of participants had a normal ECG, followed by hypertrophy (8.5%), conduction disturbance (8.2%), and ST/T change (8.0%; Table S2). The prevalence of atrial fibrillation and ventricular premature complex was 2.4% and 2.2% among all participants, respectively, with a higher prevalence in the fracture event group.

**TABLE 1:** Clinical characteristics of the study participants in the VERTE‐X cohort.

Clinical characteristics	Total (*n* = 7738)	Incident fracture		*p*
		No (*n* = 6962)	Yes (*n* = 776)	
Age, year	63.5 ± 10.4	62.9 ± 10.4	68.5 ± 8.9	< 0.001
Women, *n* (%)	4600 (59.5)	4016 (57.7)	584 (75.3)	< 0.001
BMI, kg/m^2^	24.3 ± 3.3	24.4 ± 3.2	23.7 ± 3.7	< 0.001
Previous fracture, *n* (%)	1307 (16.9)	955 (13.7)	352 (45.4)	< 0.001
Chronic glucocorticoid use, *n* (%)	294 (3.8)	225 (3.2)	69 (8.9)	< 0.001
Femoral neck BMD T‐score (*n* = 5859)	−1.5 ± 1.2	−1.5 ± 1.2	−2.2 ± 1.1	< 0.001
Charlson comorbidity index ≥ 2, *n* (%)	1209 (15.6)	976 (14.0)	233 (30.0)	< 0.001
ECG‐HD	1.40 ± 0.93	1.36 ± 0.93	1.74 ± 0.92	< 0.001
ECG parameters				
Ventricular rate, beats/min	71.9 ± 12.5	71.6 ± 12.4	73.6 ± 13.7	< 0.001
QRS duration, ms	92.3 ± 14.2	92.5 ± 14.1	90.7 ± 15.5	0.001
Corrected QT interval, ms	430.8 ± 26.8	430.0 ± 26.4	438.6 ± 29.1	< 0.001
R axis, degree	32.5 ± 34.2	32.9 ± 33.9	28.8 ± 36.3	0.001
T axis, degree	42.7 ± 28.3	42.1 ± 27.1	47.9 ± 36.7	< 0.001

*Note:* Data were presented as mean ± standard deviation or number (%).

Abbreviations: BMI, body mass index; ECG‐HD, electrocardiogram‐based homeostatic dysregulation index.

### 3.2 Association of ECG‐HD With Age

**FIGURE 3:**
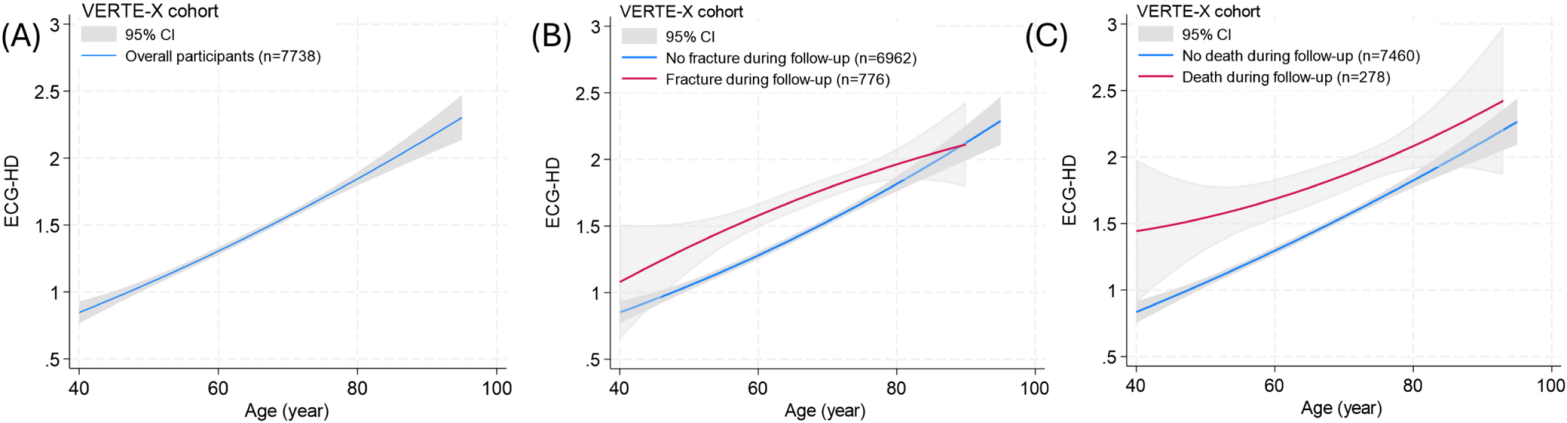
Association of ECG‐HD with age. (A) Individuals in the VERTE‐X cohort (*n* = 7738; age 40 or older) participants; (B) VERTE‐X cohort (*n* = 7738; age 40 or older) participants with (red) or without fracture events (blue) during follow‐up; (C) VERTE‐X cohort participants with (red) or without death (blue) during follow‐up.

Variability (or dispersion) of ECG parameters increased across young to old age groups (Table S1), with a notable increment in the coefficient of variation for QRS duration, R axis, and T axis (Figure S2). Reflecting this, ECG‐HD increased modestly with age (Pearson correlation coefficient 0.28, *p* < 0.001; Figures 3A and S3). Individuals with fracture or mortality events during follow‐up had elevated ECG‐HD at baseline compared to those without across ages (Figure 3B, 3C).

### 3.3 ECG‐HD According to ECG Diagnoses

In Figure S4, the mean and 95% confidence interval of ECG‐HD were plotted by categories of ECG diagnostic, rhythm, and form statements. In each diagnostic, rhythm, and form categories, individuals with any abnormal ECG statement had a higher ECG‐HD score compared to the referent group without abnormalities. Groups with multiple (two or more) ECG diagnoses had the highest ECG‐HD, followed by conduction disturbance and ST/T change. Among rhythm and form statements, individuals with sinus tachycardia, atrial fibrillation, or ventricular premature complex had a higher average ECG‐HD compared to those without.

### 3.4 Association of ECG‐HD With Fracture and Mortality

**FIGURE 4:**
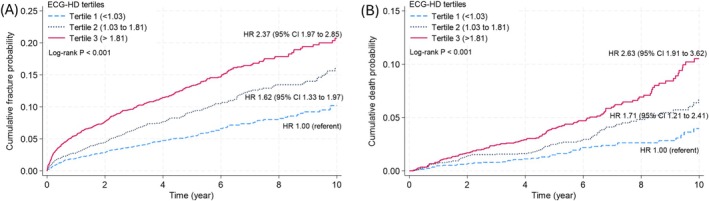
Kaplan–Meier failure curves by ECG‐HD tertiles (tertile 1: < 1.03; tertile 2: 1.03–1.81; tertile 3: > 1.81) for (A) incident fracture and (B) mortality in the VERTE‐X ECG study. CI, confidence interval; ECG‐HD, electrocardiogram‐based homeostatic dysregulation index; HR, hazard ratio.

In the VERTE‐X ECG study, the incidence of any fracture and mortality was 19.3/1000 person‐years and 6.4/1000 person‐years, respectively (Table S3). When participants were stratified into tertiles of ECG‐HD, individuals in higher ECG‐HD tertiles had a greater risk of fracture and mortality (for fracture and mortality: T3 vs. T1, hazard ratio [HR] 2.37 and 2.63; T2 vs. T1, HR 1.62 and 1.71, respectively) compared to those in the lowest ECG‐HD tertile (T1; Figure 4). The risk of incident fracture increased by 48% per one standard deviation increment of ECG‐HD (unadjusted HR 1.48, 95% CI 1.37–1.58). The association was attenuated but remained significant after adjustment for age (adjusted HR [aHR] 1.30, 95% CI 1.21–1.40, Table 2) and it remained robust and significant (aHR 1.28, 95% CI 1.15–1.42) after further adjustments for clinical risk factors, ECG diagnoses, and femoral neck BMD (Tables 2 and S4). Elevated ECG‐HD was also associated with a greater risk of fractures by sites (vertebral, nonvertebral, major osteoporotic, and hip).

**TABLE 2:** Association of ECG‐HD with incident fracture and mortality in the VERTE‐X cohort.

HR per 1 SD increment of ECG‐HD	Model 1 (unadjusted)		Model 2 (adjusted for age)		Model 3 (model 2 + clinical risk factors^a^ and ECG statements^b^)		Model 4 (model 3 + FNBMD, *n* = 5859)	
Outcomes	Unadjusted HR (95% CI)	*p*	Adjusted HR (95% CI)	*p*	Adjusted HR (95% CI)	*p*	Adjusted HR (95% CI)	*p*
Any fracture^c^	1.48 (1.37–1.58)	< 0.001	1.30 (1.21–1.40)	< 0.001	1.24 (1.14–1.35)	< 0.001	1.28 (1.15–1.42)	< 0.001
Vertebral	1.37 (1.26–1.50)	< 0.001	1.22 (1.11–1.33)	< 0.001	1.18 (1.06–1.31)	0.002	1.24 (1.09–1.41)	0.001
Nonvertebral	1.57 (1.42–1.75)	< 0.001	1.38 (1.22–1.53)	< 0.001	1.28 (1.13–1.45)	< 0.001	1.30 (1.12–1.53)	0.001
MOF	1.42 (1.31–1.53)	< 0.001	1.24 (1.15–1.35)	< 0.001	1.20 (1.10–1.32)	< 0.001	1.25 (1.11–1.40)	< 0.001
Hip	1.74 (1.47–2.07)	< 0.001	1.40 (1.17–1.68)	< 0.001	1.35 (1.10–1.65)	0.004	1.32 (1.02–1.71)	0.035
Mortality	1.62 (1.44–1.82)	< 0.001	1.43 (1.27–1.62)	< 0.001	1.34 (1.16–1.55)	< 0.001	1.44 (1.18–1.74)	< 0.001

Abbreviations: ECG‐HD, electrocardiogram‐based homeostatic dysregulation index; HR, hazard ratio; MOF, major osteoporotic fracture (wrist, spine, hip, and humerus); SD, standard deviation.

^a^ Clinical risk factors: sex, body mass index, previous fracture, and chronic glucocorticoid use.

^b^ ECG statements: Diagnostics (6 categories; normal ECG [referent], conduction disturbance, hypertrophy, myocardial infarction, ST/T change, and two or more abnormalities), rhythm (5 categories; normal sinus rhythm [referent], sinus bradycardia, sinus tachycardia, atrial fibrillation, and other), and form (4 categories; normal form [referent], non‐specific St changes, ventricular premature complex, and other).

^c^ Any fracture: incident fractures at spine (morphologic or clinical), hip, wrist, humerus, pelvis, and lower legs.

ECG‐HD was also associated with the risk of mortality (Table 2; unadjusted HR 1.62 per one standard deviation increment of ECG‐HD, 95% CI 1.44–1.82) that was attenuated but remained statistically significant after adjustment for age (aHR 1.43, 95% CI 1.27–1.62) and was unchanged by further adjustments for clinical risk factors and ECG diagnoses (aHR 1.44, 95% CI 1.18–1.74). The association between ECG‐HD and outcomes persisted even after including Charlson comorbidity index at baseline in the fully adjusted model 4 (for fracture, aHR 1.25, 95% CI 1.12–1.38, *p* < 0.001; for mortality, aHR 1.35, 95% CI 1.11–1.63, *p* = 0.002). In sensitivity analyses, changing the centroid of ECG‐HD to the mean of the overall reference population did not alter the association (for fracture: aHR 1.26, 95% CI 1.13–1.41; for mortality: aHR 1.37, 95% CI 1.13–1.67). The association between ECG‐HD and outcomes remained independent when the analyses were limited to individuals without any prevalent vertebral fracture identified by ABQ method at the index date (Table S5). The association remained robust when the analysis was further limited to individuals without any previous fracture including morphologic vertebral or clinical nonvertebral fractures (for overall fracture, aHR 1.34, 95% CI 1.16–1.55, *p* < 0.001; for mortality, aHR 1.55, 95% CI 1.18–2.04, *p* = 0.002). ECG‐HD showed a stronger association with incident fracture and mortality when compared to other ECG measurements (Table S6). After accounting for the competing risk of death in Fine and Gray model with adjustment for covariates in multivariable model 4, ECG‐HD remained significantly associated with an increased risk of fracture (subdistribution hazard ratio [SHR] = 1.20, 95% CI 1.07–1.35, *p* = 0.003).

## 4 Discussion

We found that the degree of entropy in cardiac function, as measured by ECG‐HD, predicted not only mortality but also incident fracture, an outcome in a different physiological system. These findings support the hypothesis that the degree of disorder—entropy—that accumulates with aging in one system would be associated with the same process in other systems, with the consequences of failure—fracture in one and mortality involving entropy in other systems. As expected, the measurement of entropy, *D*_*M*_ in ECGs, increased with age. As predicted for a fundamental process of aging, the association of entropy in the ECG with an increased risk of fracture was attenuated but remained significant after adjustment for calendar age. It also remained strong and statistically significant after adjustments for the established physiological association of BMD with fracture risk. The association was also independent of fracture history.

Similarly, the results support the hypothesis that accumulating disorder in the cardiac conducting system, assessed by variability in ECG measurements, would be associated with multisystem entropy and increased risk of mortality. As expected, the association was attenuated but remained strong and significant with adjustments for age and was not attributable to medical or physiological abnormalities assessed by ECG.

Previous studies that quantified *D*_*M*_ using blood biomarkers found that *D*_*M*_, reflecting physiological dysregulation of systems, increased with age and predicted adverse health outcomes, including mortality, frailty, diabetes, and heart disease (Cohen et al. 2013; Li et al. 2015; Cohen et al. 2014). Notably, the findings were not highly sensitive to which biomarkers were used (Li et al. 2015). Correlations between dysregulation levels across systems were weak but significant, suggesting a fundamental process of increasing disorder with age across a set of system‐specific processes—namely, entropy (Li et al. 2015). We extended these findings to ECG measurements to assess the degree of disorder in the cardiac conduction system. This was strongly associated with failure in other systems, fractures, and overall mortality. These findings could open the possibility of investigating a multi‐modal *D*_*M*_ to measure the entropy of aging in different systems, at different biological scales, and with different data modalities.

As an observational study, this analysis cannot establish that entropy caused fractures or mortality. Entropy is difficult to conceptualize and measure directly because it manifests at levels ranging from molecular to tissue to whole‐organism and essentially represents random disorder (Bortz 2nd 1986). It does not have a readily recognizable characteristic such as the density of bone or contractility of the heart. *D*_*M*_ is a statistical property expected of entropy: increasing disorder of a geometric system with aging (Cohen et al. 2013; Li et al. 2015). Entropy is not limited to a single component of a system so that, as expected, all components of the ECG contributed to the summary measurement of *D*_*M*_ that was the strongest predictor of fracture and mortality.

This study has an important strength that the ECGs were taken as a routine part of health screening in South Korea. This minimizes a potential bias that a potential confounding health factor led to the selection of patients who had ECGs and also increased the risk of fracture. Similarly, BMD was obtained as a screening test so that this key covariate is not likely to be confounded by an underlying health impairment. The study has sufficient size and duration to find important associations that remain robust to adjustment for covariates. It has a limitation that there were insufficient numbers of individual fractures and causes of death to test whether *D*_*M*_ was differentially associated with certain types of fractures or causes of death. As a limitation of the hospital‐based study, outcomes were defined based on death certificates issued in the institution, which may have led to incomplete ascertainment of outcomes during follow‐up. The health records do not include routinely ascertained histories of falls, so the analysis could not explore the associations of *D*_*M*_ with the risk of falling or whether the association of *D*_*M*_ with fracture was partially mediated by an increased risk of fractures. *D*_*M*_ as a measure of entropy was only measured in the cardiac system. Similar observations with other aging‐related tissue systems could be possible, which merit further investigation. Information on lifestyle factors and medications was not available in this study.

In summary, this study raises the possibility that associations of adverse outcomes of aging, including fracture and mortality, are partly due to the fundamental process of increasing molecular disorder with aging. We have applied a method for quantifying entropy that is applicable to many other images and types of data. Additional applications of this and other approaches to quantifying entropy to other cohorts and populations for other aging‐related outcomes would contribute to understanding the potential role of entropy in aging and its consequences. Future work should explore integrated and/or multi‐modal approaches to entropy measurement and their predictive power for a wide range of health outcomes.

## Author Contributions

**Namki Hong:** conceptualization, methodology, formal analysis, investigation, data curation, writing – original draft, visualization, writing‐review and editing. **Sang Wouk Cho:** software, methodology, formal analysis, investigation, visualization, writing – review and editing. **Jungheui Kim:** methodology, formal analysis, investigation, data curation. **Hanjin Park:** writing – review and editing, resources, data curation. **Dong‐Seon Kang:** writing – review and editing, resources, data curation. **Seng Chan You:** writing – review and editing, supervision, resources, data curation. **Hee Tae Yu:** methodology, formal analysis, investigation, data curation, writing – review and editing. **Kyoung Min Kim:** writing – review and editing, resources, data curation. **Yumie Rhee:** writing – review and editing, supervision. **Alan A. Cohen:** conceptualization, methodology, writing – review and editing, supervision. **Steven R. Cummings:** conceptualization, project administration, investigation, resources, methodology, writing – original draft, writing – review and editing. All authors have read and finally agreed to the published version of the manuscript.

## Conflicts of Interest

The authors declare no conflicts of interest.

## Supporting information


**Data S1:** acel70227‐sup‐0001‐DataS1.pdf.

## Data Availability

The data that support the findings of this study are available from the corresponding author upon reasonable request and with appropriate approvals.
