# Supplementary material for: Entropy, Assessed by Homeostatic Dysregulation on Electrocardiograms Predicts Fracture and Mortality
Source: Aging Cell. 2025 Sep 10;24(12):e70227. doi: 10.1111/acel.70227 (PMC12686576; doi:10.1111/acel.70227)
Supplement: Supplementary file 1 — Data S1: acel70227‐sup‐0001‐DataS1.pdf. [file ACEL-24-e70227-s001.pdf]

Supplementary materials

Entropy, assessed by homeostatic dysregulation on electrocardiograms predicts fracture and mortality

Namki Hong[1,2,3], Sang Wouk Cho[4,5], Jungheui Kim[4,5], Hanjin Park[6], Dong-Seon Kang[6], Seng Chan You[4,5], Hee Tae Yu[6], Kyoung Min Kim[7], Yumie Rhee[1], Alan A. Cohen[8], and Steven R. Cummings[2,3]

Corresponding author: Namki Hong

[1] Division of Endocrinology, Department of Internal Medicine, Severance Hospital, Yonsei University College of Medicine, Seoul, South Korea

[2] San Francisco Coordinating Center, California Pacific Medical Center Research Institute, San Francisco, CA, USA

[3] Department of Epidemiology and Biostatistics, University of California, San Francisco, San Francisco, CA, USA

[4] Institute for Innovation in Digital Healthcare (IIDH), Yonsei University Health System, Seoul, Korea

[5] Department of Biomedical Systems Informatics, Yonsei University, College of Medicine, Seoul, South Korea

[6] Division of Cardiology, Department of Internal Medicine, Severance Hospital, Yonsei University College of Medicine, Seoul, South Korea

[7] Division of Endocrinology, Department of Internal Medicine, Yongin Severance Hospital, Yonsei University College of Medicine, Yongin, South Korea

[8] Department of Environmental Health Sciences, Butler Columbia Aging Center, Mailman School of Public Health, Columbia University, New York, NY, USA

Supplementary table 1. ECG parameters by age groups in the checkup ECG study (n=37848)

Age groups	Overall	19-29	30-39	40-49	50-59	60-69	70 or older
Women	N=17683	N=1643	N=4389	N=4803	N=4480	N=1986	N=562
ECG-HD	1.31 ± 0.87	1.08 ± 0.86	1.08 ± 0.88	1.24 ± 0.88	1.46 ± 0.87	1.70 ± 0.90	1.94 ± 0.83
Ventricular rate, beats/min	67.1 ± 10.8	68.3 ± 11.2	68.2 ± 10.9	66.8 ± 10.4	65.7 ± 10.7	66.7 ± 11.2	68.2 ± 10.2
QRS duration, ms	88.1 ± 9.8	87.1 ± 8.6	87.4 ± 8.7	87.6 ± 9.3	88.8 ± 10.1	89.6 ± 11.8	89.0 ± 13.5
Corrected QT interval, ms	429.7 ± 22.5	421.9 ± 22.0	426.3 ± 20.9	430.5 ± 22.0	431.8 ± 22.4	435.1 ± 24.0	438.1 ± 23.3
R axis, degree	52.8 ± 30.7	69.4 ± 24.9	64.5 ± 26.0	54.4 ± 28.7	45.1 ± 30.3	34.3 ± 29.3	25.4 ± 33.5
T axis, degree	40.8 ± 20.5	42.3 ± 18.2	41.8 ± 18.4	40.6 ± 19.1	40.2 ± 21.7	39.4 ± 25.1	39.3 ± 24.5
Men	N=19985	N=1071	N=4681	N=5875	N=5458	N=2330	N=570
ECG-HD	1.30 ± 0.88	1.08 ± 0.81	1.06 ± 0.84	1.22 ± 0.85	1.41 ± 0.92	1.61 ± 0.92	1.97 ± 1.04
Ventricular rate, beats/min	66.6 ± 10.9	67.2 ± 11.8	67.5 ± 10.8	66.8 ± 10.5	65.7 ± 10.7	66.3 ± 11.9	66.9 ± 12.7
QRS duration, ms	96.8 ± 11.9	97.0 ± 10.6	96.6 ± 10.6	96.1 ± 11.2	97.1 ± 12.5	97.6 ± 13.8	98.3 ± 16.5
Corrected QT interval, ms	415.5 ± 22.3	404.4 ± 22.0	411.1 ± 21.3	415.3 ± 21.2	418.3 ± 22.0	420.9 ± 23.3	426.5 ± 25.1
R axis, degree	44.2 ± 34.8	62.3 ± 30.6	53.6 ± 32.5	46.1 ± 33.1	37.3 ± 34.7	32.9 ± 36.0	26.7 ± 38.3
T axis, degree	39.9 ± 22.3	41.7 ± 17.6	39.7 ± 19.3	39.3 ± 21.8	39.0 ± 23.1	42.5 ± 26.6	43.5 ± 27.8

Data were presented as mean ± standard deviation (coefficient of variation). Abbreviations: ECG-HD, electrocardiogram-based homeostatic dysregulation index.

Supplementary table 2. ECG statements of study participants in the VERTE-X ECG study

ECG statements	Total (n=7738)	Incident fracture		P-value
		No (n=6962)	Yes (n=776)	
Diagnostic, n (%)				<0.001
Normal ECG	5262 (68.0)	4785 (68.7)	477 (61.5)	
Conduction disturbance	632 (8.2)	567 (8.1)	65 (8.4)	
Hypertrophy	660 (8.5)	589 (8.5)	71 (9.2)	
Myocardial infarction	181 (2.3)	162 (2.3)	19 (2.4)	
ST/T change	619 (8.0)	528 (7.6)	91 (11.7)	
Two or more abnormalities	384 (5.0))	331 (4.8)	53 (6.8)	
Rhythm, n (%)				<0.001
Normal sinus rhythm	6544 (84.6)	5910 (84.9)	634 (81.7)	
Sinus bradycardia	628 (8.1)	574 (8.2)	54 (7.0)	
Sinus tachycardia	181 (2.4)	146 (2.1)	35 (4.5)	
Atrial fibrillation	189 (2.4)	153 (2.2)	36 (4.6)	
Other	196 (2.5)	179 (2.6)	17 (2.2)	
Form, n (%)				<0.001
Normal form	4814 (62.2)	4384 (63.0)	430 (55.4)	
Non-specific ST changes	270 (3.5)	220 (3.2)	50 (6.4)	
Ventricular premature complex	172 (2.2)	146 (2.0)	26 (3.4)	
Other	2482 (32.1)	2212 (31.8)	270 (34.8)	

Abbreviations: ECG, electrocardiogram.

Supplementary table 3. Incidence rate of outcomes in the VERTE-X ECG study

Outcomes	Event n (%)	Incidence rate (/ 1000 person-years)
Any fracture**	776 (10.0)	19.3
Vertebral	514 (6.6)	12.8
Nonvertebral	345 (4.5)	8.2
MOF	647 (8.4)	16.5
Hip	132 (1.7)	3.1
Mortality	278 (3.6)	6.4

**Any fracture: incident fractures at spine (morphologic or clinical), hip, wrist, humerus, pelvis, and lower legs.
Abbreviations: MOF, major osteoporotic fracture (wrist, spine, hip, and humerus).

Supplementary table 4. Cox proportional hazard models to predict incident fracture in the VERTE-X ECG study

	Univariate model		Multivariable model adjusted for clinical risk factors, ECG statements, and femoral neck BMD (n=5859)	
Predictors	Unadjusted HR (95 % CI)	P-value	Adjusted HR (95 % CI)	P-value
ECG-HD, per 1 SD increment	1.48 (1.37 to 1.58)	<0.001	1.28 (1.15 to 1.42)	<0.001
Age, per 1 year increment	1.06 (1.05 to 1.07)	<0.001	1.02 (1.01 to 1.04)	<0.001
Women	1.94 (1.65 to 2.29)	<0.001	1.14 (0.92 to 1.42)	0.223
BMI	0.93 (0.91 to 0.95)	<0.001	0.96 (0.93 to 0.98)	0.005
Previous fracture	4.79 (4.16 to 5.52)	<0.001	3.10 (2.55 to 3.76)	<0.001
Chronic glucocorticoid use	2.19 (1.71 to 2.81)	<0.001	2.00 (1.49 to 2.67)	<0.001
ECG statements				
Diagnostic				
Normal ECG	1.00 (referent)		1.00 (referent)	
Conduction disturbance	1.14 (0.88 to 1.48)	0.314	0.93 (0.66 to 1.30)	0.669
Hypertrophy	1.23 (0.96 to 1.58)	0.094	1.07 (0.75 to 1.52)	0.708
Myocardial infarction	1.18 (0.74 to 1.87)	0.474	0.61 (0.30 to 1.24)	0.170
ST/T change	1.62 (1.29 to 2.02)	<0.001	1.10 (0.78 to 1.54)	0.606
Two or more abnormalities	1.55 (1.17 to 2.06)	0.002	0.90 (0.61 to 1.34)	0.597
Rhythm, n (%)				
Normal sinus rhythm	1.00 (referent)		1.00 (referent)	
Sinus bradycardia	0.86 (0.65 to 1.13)	0.299	0.80 (0.56 to 1.14)	0.227
Sinus tachycardia	2.12 (1.51 to 2.98)	<0.001	1.39 (0.92 to 2.10)	0.113
Atrial fibrillation	2.04 (1.46 to 2.86)	<0.001	1.04 (0.67 to 1.62)	0.841
Other	0.84 (0.52 to 1.36)	0.479	0.60 (0.33 to 1.10)	0.100
Form, n (%)				
Normal form	1.00 (referent)		1.00 (referent)	
Non-specific ST changes	2.10 (1.57 to 1.46)	<0.001	1.09 (0.71 to 1.68)	0.670
Ventricular premature complex	1.82 (1.23 to 2.71)	0.003	0.97 (0.58 to 1.62)	0.928
Other	1.25 (1.23 to 2.71)	0.004	0.94 (0.73 to 1.20)	0.639
Femoral neck T-score, per 1 unit decrement	1.70 (1.57 to 1.85)	<0.001	1.25 (1.14 to 1.38)	<0.001

Supplementary table 5. Association of ECG-HD with outcomes in participants without prevalent vertebral fracture at the index date (n=6569) in the VERTE-X ECG study

HR per 1 SD increment of ECG-HD	Model 1 (unadjusted)		Model 2 (adjusted for age)		Model 3 (model 2 + clinical risk factors* and ECG statements†)		Model 4 (model 3 + FNBMD, n=4979)	
Outcomes	Unadjusted HR (95% CI)	P-value	Adjusted HR (95% CI)	P-value	Adjusted HR (95% CI)	P-value	Adjusted HR (95% CI)	P-value
Any fracture**	1.48 (1.35 to 1.61)	<0.001	1.32 (1.21 to 1.45)	<0.001	1.21 (1.09 to 1.35)	<0.001	1.26 (1.10 to 1.43)	0.001
Vertebral	1.43 (1.28 to 1.59)	<0.001	1.29 (1.15 to 1.45)	<0.001	1.23 (1.08 to 1.40)	0.002	1.31 (1.11 to 1.53)	0.001
Nonvertebral	1.50 (1.30 to 1.72)	<0.001	1.35 (1.17 to 1.55)	<0.001	1.19 (1.01 to 1.39)	0.034	1.20 (0.98 to 1.47)	0.085
MOF	1.44 (1.30 to 1.58)	<0.001	1.28 (1.16 to 1.42)	<0.001	1.21 (1.08 to 1.36)	0.001	1.29 (1.12 to 1.50)	0.001
Hip	1.80 (1.41 to 2.31)	<0.001	1.54 (1.20 to 2.00)	0.001	1.53 (1.13 to 2.08)	0.006	1.67 (1.08 to 2.58)	0.020
Mortality	1.71 (1.47 to 1.98)	<0.001	1.58 (1.35 to 1.85)	<0.001	1.53 (1.28 to 1.85)	<0.001	1.58 (1.21 to 2.05)	0.001

*Clinical risk factors: sex, body mass index, previous fracture, and chronic glucocorticoid use
†ECG statements: Diagnostics (6 categories; normal ECG [referent], conduction disturbance, hypertrophy, myocardial infarction, ST/T change, and two or more abnormalities), rhythm (5 categories; normal sinus rhythm [referent], sinus bradycardia, sinus tachycardia, atrial fibrillation, and other), and form (4 categories; normal form [referent], non-specific St changes, ventricular premature complex, and other).
Abbreviations: ECG-HD, electrocardiogram-based homeostatic dysregulation index; SD, standard deviation; HR, hazard ratio; MOF, major osteoporotic fracture (wrist, spine, hip, and humerus).
**Any fracture: incident fractures at spine (morphologic or clinical), hip, wrist, humerus, pelvis, and lower legs.

Supplementary table 6. Association of ECG parameters with incident fracture and mortality in the VERTE-X ECG cohort

HR per 1 SD increment	Model 1 (unadjusted)		Model 2 (adjusted for age)		Model 3 (model 2 + clinical risk factors* and ECG statements†)		Model 4 (model 3 + FNBMD, n=5859)	
Predictor#	Unadjusted HR (95% CI)	P-value	Adjusted HR (95% CI)	P-value	Adjusted HR (95% CI)	P-value	Adjusted HR (95% CI)	P-value
	Outcome: Any fracture**							
ECG-HD	1.48 (1.37 to 1.58)	<0.001	1.30 (1.21 to 1.40)	<0.001	1.24 (1.14 to 1.35)	<0.001	1.28 (1.15 to 1.42)	<0.001
Ventricular rate	1.14 (1.07 to 1.21)	<0.001	1.12 (1.06 to 1.19)	<0.001	1.02 (0.95 to 1.11)	0.483	1.01 (0.92 to 1.11)	0.754
QRS duration	0.98 (0.93 to 1.03)	0.511	0.97 (0.92 to 1.15)	0.180	0.99 (0.94 to 1.04)	0.833	1.01 (0.95 to 1.07)	0.759
Corrected QT interval	1.18 (1.12 to 1.24)	<0.001	1.11 (1.05 to 1.17)	<0.001	1.07 (1.02 to 1.14)	0.013	1.02 (0.95 to 1.10)	0.551
R axis	0.86 (0.81 to 0.91)	<0.001	0.95 (0.89 to 1.00)	0.057	0.95 (0.89 to 1.01)	0.086	0.95 (0.88 to 1.01)	0.118
T axis	1.11 (1.07 to 1.16)	<0.001	1.08 (1.04 to 1.12)	<0.001	1.05 (1.01 to 1.09)	0.003	1.04 (0.98 to 1.08)	0.146
	Outcome: mortality							
ECG-HD	1.62 (1.44 to 1.82)	<0.001	1.43 (1.27 to 1.62)	<0.001	1.34 (1.16 to 1.55)	<0.001	1.44 (1.18 to 1.74)	<0.001
Ventricular rate	1.34 (1.23 to 1.47)	<0.001	1.32 (1.21 to 1.45)	<0.001	1.17 (1.04 to 1.31)	0.008	1.27 (1.09 to 1.49)	0.002
QRS duration	0.99 (0.91 to 1.07)	0.848	0.98 (0.90 to 1.06)	0.549	1.01 (0.93 to 1.09)	0.896	1.06 (0.96 to 1.17)	0.281
Corrected QT interval	1.28 (1.19 to 1.39)	<0.001	1.22 (1.13 to 1.33)	<0.001	1.10 (1.01 to 1.21)	0.036	1.17 (1.04 to 1.31)	0.007
R axis	0.97 (0.88 to 1.06)	0.470	1.07 (0.97 to 1.17)	0.167	0.98 (0.89 to 1.08)	0.723	0.90 (0.79 to 1.02)	0.106
T axis	1.15 (1.09 to 1.22)	<0.001	1.12 (1.06 to 1.19)	<0.001	1.08 (1.02 to 1.14)	0.005	1.06 (0.98 to 1.15)	0.093

Hazard ratios per 1 standard deviation increment of ECG parameters.

Standardized ECG parameters were entered into multivariable models separately.

*Clinical risk factors: sex, body mass index, previous fracture, and chronic glucocorticoid use

†ECG statements: Diagnostics (6 categories; normal ECG [referent], conduction disturbance, hypertrophy, myocardial infarction, ST/T change, and two or more abnormalities), rhythm (5 categories; normal sinus rhythm [referent], sinus bradycardia, sinus tachycardia, atrial fibrillation, and other), and form (4 categories; normal form [referent], non-specific St changes, ventricular premature complex, and other).

Abbreviations: ECG-HD, electrocardiogram-based homeostatic dysregulation index; SD, standard deviation; HR, hazard ratio; MOF, major osteoporotic fracture (wrist, spine, hip, and humerus).

**Any fracture: incident fractures at spine (morphologic or clinical), hip, wrist, humerus, pelvis, and lower legs.

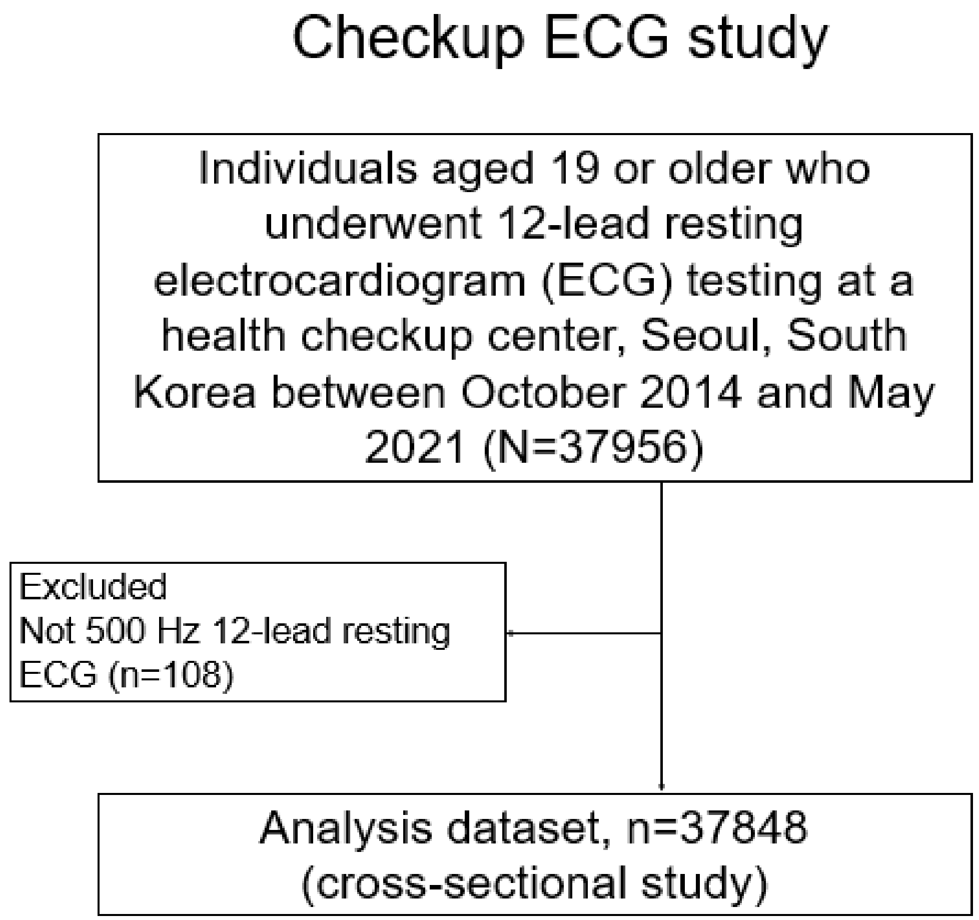

Supplementary figure 1. Checkup ECG study to derive centroids to calculate ECG-homeostatic dysregulation index

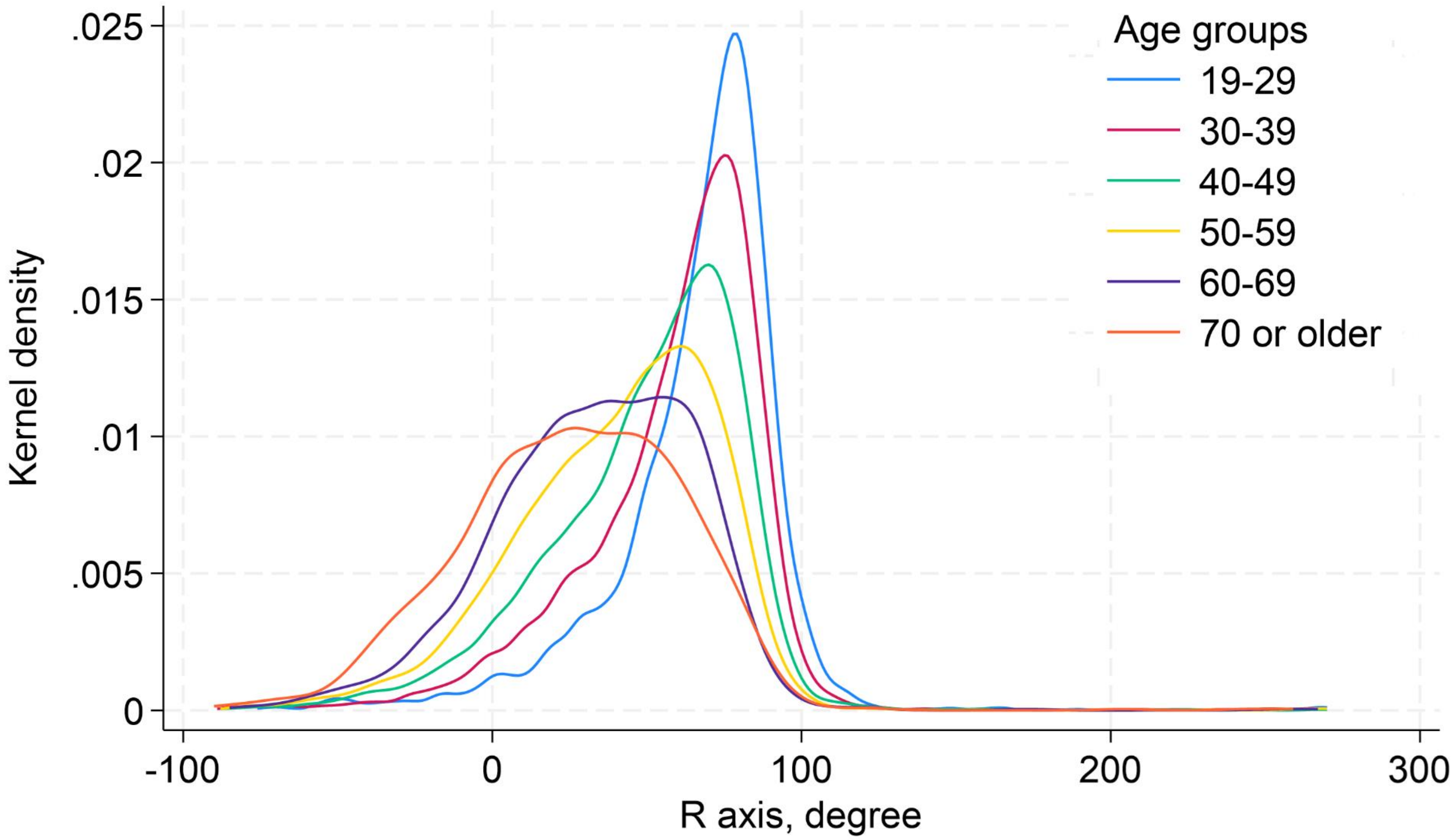

Supplementary figure 2. Probability distribution of R axis by age groups in the checkup ECG study (total n=37848; age 19-29, n=2714; age 30-39, n=9070; age 40-49, n=10678; age 50-59, n=9938; age 60-69, n=4316; age 70 or older, n=1132).

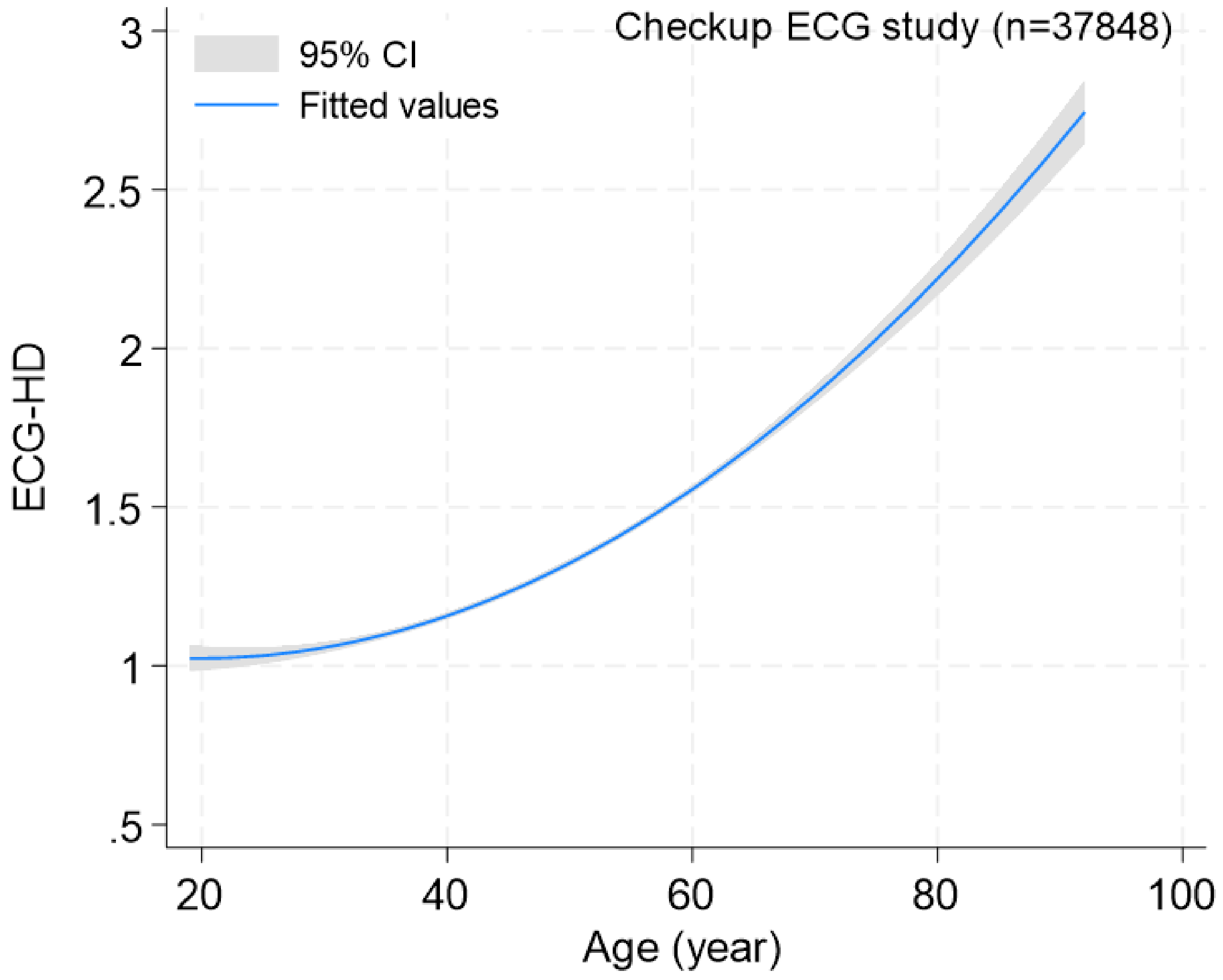

Supplementary figure 3. Association of ECG-HD with age in the checkup ECG study (n=37848)

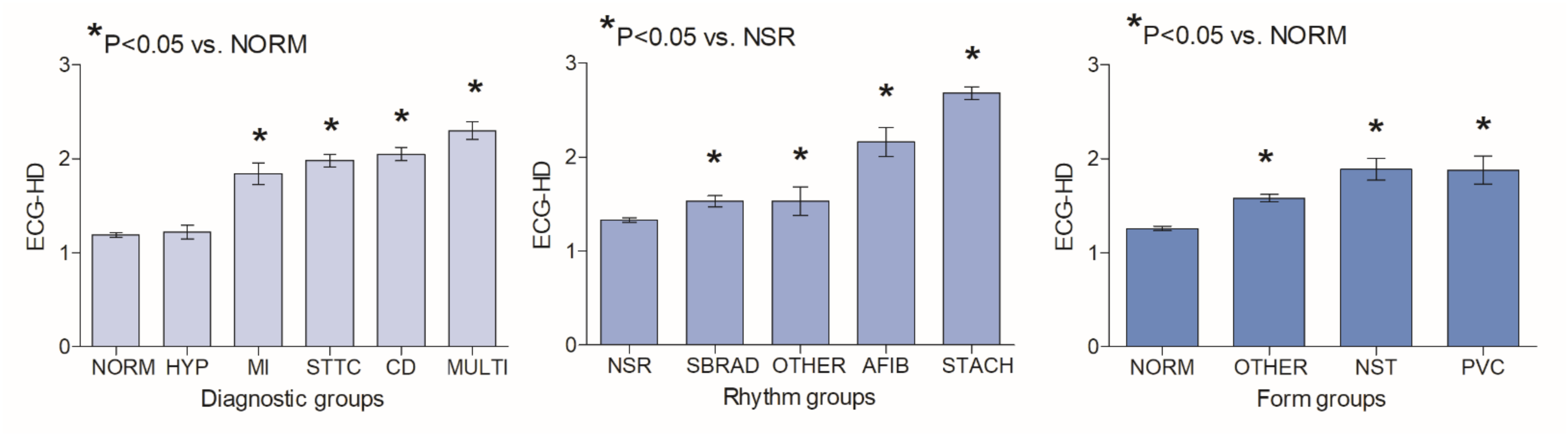

Supplementary figure 4. Mean and 95% confidence interval of ECG-HD by ECG statements ([A] diagnostic, [B] rhythm, and [C] forms) in the VERTE-X ECG study.

*: Bonferroni-corrected P values from one-way analysis of variance <0.05 compared to referent group.

Abbreviations: NORM, normal; HYP, hypertrophy; MI, myocardial infarction; STTC, ST/T change; CD, conduction disturbance; MULTI, multiple diagnostics; NSR, normal sinus rhythm; SBRAD, sinus bradycardia; OTHER, other; AFIB, atrial fibrillation; STACH, sinus tachycardia; NST, non-specific T change; PVC, premature ventricular contraction.
